# Supplementary material for: Rapid development of cloud-native intelligent data pipelines for scientific data streams using the HASTE Toolkit
Source: Gigascience. 2021 Mar 19;10(3):giab018. doi: 10.1093/gigascience/giab018 (PMC7976223; doi:10.1093/gigascience/giab018)
Supplement: giab018_Response_to_Reviewer_Comments_Original_Submission [file giab018_response_to_reviewer_comments_original_submission.pdf]

Author's Response To Reviewer Comments

Close

Dear Editor,

Please find attached our revised manuscript "Rapid development of cloud-native intelligent data pipelines for scientific data streams using the HASTE Toolkit".

We were happy to read the mostly positive comments about the manuscript, and we thank the reviewers for raising some relevant points regarding reproducibility and documentation which we have now addressed. Specifically, we have improved documentation throughout and expanded step-by-step guides to deploy the example HASTE pipelines.

Below we provide detailed responses and comments to the reviewer's remarks and describe the updates we have made in the revised version of our manuscript. We hope that you now find it suitable for publication.

Our responses to reviewers' comments are shown with a '>'.

Sincerely,

Ben Blamey and co-authors

Editor's comments:

I'd like to highlight point #2 of reviewer 2 in particular, regarding reproducibility. For a GigaScience "Technical Note", it is essential that readers can reproduce and validate results and pipelines, analyses steps, implementations etc. Please follow the reviewer's guidance, who made some suggestions in this regard. If you need a repository to store and cite data to further aid reproducibility, we can host files in our database, GigaDB. Our curators will be happy to assist you with this.

> We have followed the recommendations of reviewers and addressed improved reproducibility by expanding on and clarifying instructions for how to deploy representative examples. See detailed responses to reviewers.

Please also address the other comments of the three reviewers, e.g. the suggestion to improve documentation.

Some editorial points:

- Please register new software applications / pipelines in the bio.tools and SciCrunch.org databases to receive RRID (Research Resource Identification Initiative ID) and biotoolsID identifiers, and include these in your manuscript, in the "code availability" section. This will facilitate tracking, reproducibility and re-use of your tool.

> We have now registered the application in the bio.tools and SciCrunch.org databases and added identifiers in the manuscript (biotools:haste_toolkit, RRID:SCR_020932).

- Please also add a section "Availability of supporting source code and requirements".
> The section has been added with detailed information about versions, licences and source code.

- Please add ORCIDs of all authors to the title page, if available.
> We have now added ORCIDs for all authors. We'd be grateful for an example of how to correctly format the information in the heading with LaTeX.

Reviewer reports:

Reviewer #1: This manuscript was a pleasure to read, and from which I learned a lot. The key idea, of using "interestingness functions" to prioritise the processing, upload and storage of data (and also to direct data to cloud or edge processing) is, to excuse the pun, very interesting. The authors have done an excellent job in communicating this core idea, why this approach would be of benefit, and in presenting two real-world case studies of how interestingness functions could be used in practice. A key issue with interestingness functions is whether or not it would be possible for domain experts to write functions that would actually be useful (i.e. how could we know, a priori, whether certain data would be more interesting than others). However, when reading the case studies I saw that I was lacking imagination, and indeed there are many filters that can be applied as part of an interestingness function (e.g. focus quality, amount of debris etc). As such, I am now convinced that this approach has merit, and that this kind of smart storage / smart network / smart computing framework, whereby domain knowledge is used as a guide to prioritise and sort data into different streams, is a very good idea.

As such, the methods applied are appropriate to the aims of the study, they are very well described. and a comparison to processing without the use of interestingness functions is a suitable control (albeit the value of this paper is the presentation and proof of concept of an idea, as opposed to a real performance comparison using production software, and so such a comparison is less necessary and of lesser value).

The conclusions of the paper, namely that interestingness functions and using domain knowledge to help prioritise and sort data is supported by the data shown, and the clear arguments made in the manuscript.

The manuscript is very well written and easy to understand and follow.

There is little statistical analysis of data in the manuscript, but, as stated before, the purpose is to present an idea and proof of concept. On that topic, one issue I do have with the manuscript is that it presents the HASTE Toolkit as a complete and production-ready library. In reality, looking at the GitHub repositories associated with this manuscript, it is clear that the software is a proof of concept, and not yet ready for production deployment by others. I say this because the software lacks adequate testing (e.g. most of the test files are stubs, such as https://github.com/HASTE-project/haste-agent/blob/master/tests/test_foo.py and https://github.com/HASTE-project/cellprofiler-pipeline/blob/master/tests/test_foo.py), and there is little in the way of security analysis, robust user authentication support etc that would be needed to safely deploy this in production as a professional storage or data management product. This is not a criticism and should not delay publication of the manuscript. Just a comment that the authors need to acknowledge in the manuscript that the software is at an early stage, and a large amount of work is still needed to enable HASTE to become a robust, reliable, secure and trustable toolkit for production deployment for real research workloads.

> We agree that there are many areas that need further work in order to sustain production-critical workloads with the added security requirements they come with. We continuously improve the software, and we have already updated tests in various projects. Note that users must authenticate securely for all of the examples used in the paper. The documentation regarding user authentication has been updated (e.g. https://github.com/HASTE-project/k8s-deployments/#rabbitmq-credentials, and https://github.com/HASTE-project/haste-gateway/blob/master/readme.md)

> A paragraph explaining some caveats has been added to the end of the 'The HASTE Toolkit' section. Some known issues/enhancements are listed at https://github.com/HASTE-project/cellprofiler-pipeline/issues/2.

Reviewer #2: The paper describes an approach to processing streams of scientific data in such a way as to maximise the use of limited available compute resources (cpu, storage and network bandwidth) by grading that data by "interestingness" and prioritizing data that is more interesting.
A number of git repositories is provided which together constitute a toolkit to facilitate this approach. HASTE is the name of the toolkit, and will be used in this review also as the name for the overall approach. The paper describes the essential characteristics of the approach, and presents two case studies to demonstrate how the toolkit might work in a scientific setting.
The two case studies outline the common overall flow of HASTE, but also its flexibility in catering to different needs of a pipeline (e.g. different resource bottlenecks, and different requirements with respect to latency).

The paper is well written and presents its case in a clear and readable fashion, although some sub-sections would benefit from additional attention in this regard.
The central concept of the HASTE approach, if correctly understood, is the separation of the Interestingness Function(IF) from the subsequent Policy that uses the IF to prioritize resources.
This strikes me as a sensible application of modularity to a scientific context, and would be likely to make the toolkit adaptable to different scenarios, as indeed the case studies indicate.

Besides some minor proofreading corrections for grammar/readability which I would recommend, listed at the end, I would make two more significant observations and related recommendations, both related to reproducibility, and a final optional suggestion.

> After revising the manuscript we performed an extra proofreading for grammar and readability.

1. The body of the paper makes a good case for the IF/Policy split, but the Discussion and Conclusion sections seem undecided on what the reader should take away from the paper.
Should the reader attempt to reuse the HASTE toolkit, adapting it to their own needs? Or should they adopt the approach in the abstract, implementing it themselves?
Either (or indeed both) is acceptable, in my opinion, but the authors should be explicit in setting expectations for the reader about the viability of using the Toolkit.
In one paragraph, they describe HASTE as a "design pattern", which I think is a suitable term.

> Indeed, our intention was 'both'. We have added a comment to the conclusion to make this more clear: "Our contribution is twofold: both the HASTE pipeline model as a concept, and a Python implementation. It would be possible to re-implement the design pattern in other programming languages as needed."

But in the following paragraph, they indicate their hope that the toolkit be "bolted on" to new and existing pipelines.
This would of course be valuable, but as a reader I would have appreciated some explicit direction on "next steps" if I found the pattern and toolkit potentially applicable to my work.

> Adaptation of existing code to the HASTE Pipeline model would be a software engineering task, and the specific steps involved would depend greatly on the application in question. However, broad instructions are listed in the paper. "An existing pipeline can be adapted to use HASTE according to the following steps"

This would be a suitable outcome for a Technical Note.
The ability to use the toolkit hinges on various degrees of documentation and reproducibility.
While the code for the agent, report-generator, gateway etc are available on github, the README files appear aimed at the toolkit development team,
rather than potential users of the toolkit (or else too much is expected of those users).
If the authors intend to present the toolkit as a set of OSS resources for other researchers - which would be a worthwhile goal - it would be good to see a more
suitable documentation to guide, stepwise, those researchers.

If this is the intent for the future, rather than as part of this publication, it would be helpful if the paper were to explicitly say so.

> We have improved the documentation of various repositories with a focus on other researchers, versioning and reproducibility. In particular, the reviewers are directed to the instructions here: https://github.com/HASTE-project/k8s-deployments/blob/master/README.md

> We have tried to balance the simplicity of the system (to ease reproducibility), with the implicit complexity which comes from the configurability, features, security, scalability (etc.) to make the software usable in production environments - and this is reflected in the instructions for reproducibility.

2. With regard to reproducibility of the experiments themselves, there are a few gaps that make it difficult for a reviewer,
even one who is familiar with AWS, k8s, docker and python, to reproduce and validate the results.
Kubernetes deployment descriptors are provided for the first case study, but the instructions are not clear
> We have improved instructions with a particular focus on reproducibility.

The readme has been extensively revised to be a more ‘step by step’ instructions for reproducibility. https://github.com/HASTE-project/k8s-deployments/blob/master/README.md

...for example actual resource instances are named, which will change with each deployment) and again seem for for internal use.
> The pipeline will inevitably need to be configured to match specifics regarding e.g. storage, scaling appropriate to available resources, and generation of secure user authentication credentials. Again, the documentation has been revised extensively to make these steps clearer.

….The instructions also presuppose the existence of a k8s cluster.
> We consider deployment of Kubernetes to be outside the scope of the paper/documentation, but we have revised our documentation to provide links to deployment instructions.

...The data to be copied into the volumes is not provided.
> We have followed the instructions provided by the journal (https://academic.oup.com/gigascience/pages/technical_note “Availability of supporting data”), by publishing the datasets used in the paper in peer-reviewed data repositories. Their DOIs are cited in the paper, these are: http://doi.org/10.17044/scilifelab.12811997.v1 and https://doi.org/10.17044/scilifelab.12771614.v1 .

The use of kubernetes is intelligent, as it is a de-facto standard in cloud computing, and there is an opportunity here for
researchers to make the experiments on such a cluster reproducible by providing details instructions for their assembly (using e.g. Terraform or AWS's CloudFormation),
> See comments above re deployment of K8s.

or to provide an already-created cluster for that purpose,
and to provide the data on cloud storage that could be mapped to pods as part of the supplied kubernetes descriptors.

> Unfortunately we have no financing to provide a public-access cluster for the demonstration of the pipeline. The datasets are published as tar.gz files, a widely-supported format which makes them accessible to a wide audience regardless of their choice of cloud technology. Instructions for downloading and extracting the data are now provided in the readme.

Note that the effort in implementing point 2 above would aid in realizing point 1.

For this reason, I would urge the authors to address this issue of experimental reproducibility. A reviewer should be able to:
a. Build all Docker images from source on the command line, pushing to an image registry
b. Follow clear stepwise instructions to deploy the images to a k8s cluster (either provided, or easily created using Terraform or Cloudformation)
c. Follow clear stepwise instructions to perform salient parts of the experiment from which the data was collected.

> We have improved documentation on https://github.com/HASTE-project/k8s-deployments/blob/master/README.md to assist the user in these steps.
Also, the Docker images can be built from source, but the applications are designed to be re-usable, so that the configuration is specified separately when deploying the images.

While this is not a trivial task, the result should lead to greater interest and uptake of the proposed model.
> We agree with the reviewer and we believe that our updates to documentation has improved the system and manuscript.

3. Finally, and entirely optionally, the authors may wish to give some consideration to the suitability of Functions as a Service (aka serverless, aka lambda functions) to implement the IF.
> We added a section to the end of the conclusions detailing this.

Minor corrections and suggestions follow:

> We thank the reviewer for the careful reading. We have addressed all minor correction suggestions 1-12. For some points below we provide additional comments.

1. Standardize the use of references: Where the reference is part of the sentence, use the authors name followed by the ref number. See first para on page 2, column 2 which first references Zhang et al, but then in the following sentence just uses [9] to reference Kelleher et al. There are a number of these inconsistencies in the paper.
> Agreed, that paragraph of text of text has been revised. I've reviewed all citations in the paper, and I didn't see any others that I felt needed to change.
2. Bottom of page 3. ", or send it" should probably be ",or if to send it" (or perhaps ", or whether to send it").
> We changed this to "or if to send it".
3. Page 4, column 1, para 3: "is that users' configure" - no need for apostrophe. Done.
4. Page 6, column 1, para 2: Reword e.g. "image can have debris, can be out of focus..." etc.: Done.
5. Page 6, column 2, para 4: "which it to run" -> "which is to run". Revised wording.
6. Related to the above, the impression is given that k8s is a required component of the implementation. Apart from the issues of reproducibility of the *experimental data*, it would be good to be explicit with the reader on this matter (either here, or elsewhere in the paper).
> The deployment scripts for the example application in Case Study 1 is used with Kubernetes. None of the components of the HASTE Toolkit themselves depend on kubernetes, and this has been clarified in the paper.

7. Page 8, column 2, para 2: This section could do with a rewrite for clarity. I had difficulty understanding the meaning of "document index" in this context and this made interpretation of fig 5 problematic.
> The text has been amended/edited for clarity.
8. Page 8, column 2, final para: There appear to be two chained comparisons ("compared to", "when compared to") which renders the sentence confusing. In comparison to what is the 25% reduction in latency achieved?
> This text has been edited for clarity.
9. Page 9, caption to figure 7: "is an artifact movement over the grid" -> "is an artifact of the movement over the grid"?. Done.
10. Page 9, Conclusion: There is an orphan closing parenthesis after the word "kinds". Done.
11. Page 10, column 1, para 1: "creating an data hierarchy" -> "creating a data..." Done.
12. Page 10, column 1 para 3: "self-explainable" -> "self-explanatory"? Done.

Reviewer #3: Overall, the work presented here represents an interesting approach to processing prioritization in biological imaging experiments, a generally neglected area. The work is well explained and is worthy of publication. I find particularly valuable the idea of separating out the interestingness function (IF) from the rest of the workflow, with the idea that the domain experts (biologists) can best assess what features make an image most interesting, and that the rest of the workflow can then be configured by a computational specialist able to prioritize and configure the (many!) separate moving parts of this workflow. Allowing the domain expert a particular place in the workflow to create systems that replicate their expertise can only lead to improved processing. Overall, it is a clever hybrid strategy and I can imagine many useful cases for it.

My major concern with the work presented (which is not a publication concern, but a longevity/usefulness concern) is that, while sufficiently documented for experts and "tinkerers", it is woefully under-documented for anyone who is not. The GitHub organization contains a variety of repositories, and it is not at all clear without reading this paper how they are meant to fit together with each other or with other packages; even after reading this paper, it is not clear how one would do any other activity besides those outlined in the authors case studies. 0n serve as the interestingness function- most domain experts would need significant guidance on how to turn their expertise into a function that fulfills the requirements of the HASTE system, and very little is provided. I do not think the work must be held for publication until this documentation appears, since it appears to be sufficiently documented for an expert to begin configuration, but it would be recommendation to the authors to prioritize such work.

> These are very important suggestions. In preparing this revision we have put effort in updating the documentation of various components especially for end-users and reproducibility (see response to other reviewers) but we agree that it is important to simplify the process of adapting to new scenarios and we will put continuous efforts in this as the project continues.

Minor issue:
The authors state they use HasteStorageClient v0.13- their GH repo only reflects versions up to 0.10, indicating either commits are not being shared online or the authors need to be more careful with their versioning.
> Thank you for noticing this. The issue was that versions were bumped in setup.py, but these commits were not tagged, this has now been fixed. We have reviewed versioning and tagging on all the repositories to improve reproducibility.

Close
