# Supplementary material for: Rapid development of cloud-native intelligent data pipelines for scientific data streams using the HASTE Toolkit
Source: Gigascience. 2021 Mar 19;10(3):giab018. doi: 10.1093/gigascience/giab018 (PMC7976223; doi:10.1093/gigascience/giab018)
Supplement: giab018_Reviewer_1_Report_Original_Submission — Christopher Woods -- 10/2/2020 Reviewed [file giab018_reviewer_1_report_original_submission.pdf]

Reviewer Report

Title: Rapid development of cloud-native intelligent data pipelines for scientific data streams using the HASTE Toolkit

Version: Original Submission **Date:** 10/2/2020

Reviewer name: Christopher Woods

Reviewer Comments to Author:

This manuscript was a pleasure to read, and from which I learned a lot. The key idea, of using "interestingness functions" to prioritise the processing, upload and storage of data (and also to direct data to cloud or edge processing) is, to excuse the pun, very interesting. The authors have done an excellent job in communicating this core idea, why this approach would be of benefit, and in presenting two real-world case studies of how interestingness functions could be used in practice. A key issue with interestingness functions is whether or not it would be possible for domain experts to write functions that would actually be useful (i.e. how could we know, a priori, whether certain data would be more interesting than others). However, when reading the case studies I saw that I was lacking imagination, and indeed there are many filters that can be applied as part of an interestingness function (e.g. focus quality, amount of debris etc). As such, I am now convinced that this approach has merit, and that this kind of smart storage / smart network / smart computing framework, whereby domain knowledge is used as a guide to prioritise and sort data into different streams, is a very good idea.
As such, the methods applied are appropriate to the aims of the study, they are very well described. and a comparison to processing without the use of interestingness functions is a suitable control (albeit the value of this paper is the presentation and proof of concept of an idea, as opposed to a real performance comparison using production software, and so such a comparison is less necessary and of lesser value).
The conclusions of the paper, namely that interestingness functions and using domain knowledge to help prioritise and sort data is supported by the data shown, and the clear arguments made in the manuscript.
The manuscript is very well written and easy to understand and follow.
There is little statistical analysis of data in the manuscript, but, as stated before, the purpose is to present an idea and proof of concept. On that topic, one issue I do have with the manuscript is that it presents the HASTE Toolkit as a complete and production-ready library. In reality, looking at the GitHub repositories associated with this manuscript, it is clear that the software is a proof of concept, and not yet ready for production deployment by others. I say this because the software lacks adequate testing (e.g. most of the test files are stubs, such as https://github.com/HASTE-project/haste-agent/blob/master/tests/test_foo.py and https://github.com/HASTE-project/cellprofiler-pipeline/blob/master/tests/test_foo.py), and there is little in the way of security analysis, robust user authentication support etc that would be needed to safely deploy this in production as a professional storage or data management product. This is not a criticism and should not delay publication of the manuscript. Just a comment that the authors need to acknowledge in the manuscript that the software

is at an early stage, and a large amount of work is still needed to enable HASTE to become a robust, reliable, secure and trustable toolkit for production deployment for real research workloads. Ideally, in the abstract the authors should edit the first sentence to read; "This paper introduces the HASTE Toolkit, a proof-of-concept cloud-native software toolkit capable of partitioning data streams in order to prioritize usage of limited resources."

Level of Interest

Please indicate how interesting you found the manuscript: Choose an item.

Quality of Written English

Please indicate the quality of language in the manuscript: Choose an item.

Declaration of Competing Interests

Please complete a declaration of competing interests, considering the following questions:

- Have you in the past five years received reimbursements, fees, funding, or salary from an organisation that may in any way gain or lose financially from the publication of this manuscript, either now or in the future?
- Do you hold any stocks or shares in an organisation that may in any way gain or lose financially from the publication of this manuscript, either now or in the future?
- Do you hold or are you currently applying for any patents relating to the content of the manuscript?
- Have you received reimbursements, fees, funding, or salary from an organization that holds or has applied for patents relating to the content of the manuscript?
- Do you have any other financial competing interests?
- Do you have any non-financial competing interests in relation to this paper?

If you can answer no to all of the above, write 'I declare that I have no competing interests' below. If your reply is yes to any, please give details below.

I declare that I have no competing interests

I agree to the open peer review policy of the journal. I understand that my name will be included on my report to the authors and, if the manuscript is accepted for publication, my named report including any attachments I upload will be posted on the website along with the authors' responses. I agree for my report to be made available under an Open Access Creative Commons CC-BY license (http://creativecommons.org/licenses/by/4.0/). I understand that any comments which I do not wish to

be included in my named report can be included as confidential comments to the editors, which will not be published.

Choose an item.

To further support our reviewers, we have joined with Publons, where you can gain additional credit to further highlight your hard work (see: https://publons.com/journal/530/gigascience). On publication of this paper, your review will be automatically added to Publons, you can then choose whether or not to claim your Publons credit. I understand this statement.

Yes Choose an item.
