# Supplementary material for: Rapid development of cloud-native intelligent data pipelines for scientific data streams using the HASTE Toolkit
Source: Gigascience. 2021 Mar 19;10(3):giab018. doi: 10.1093/gigascience/giab018 (PMC7976223; doi:10.1093/gigascience/giab018)
Supplement: giab018_Reviewer_2_Report_Original_Submission — Brendan Lawlor -- 10/6/2020 Reviewed [file giab018_reviewer_2_report_original_submission.pdf]

Reviewer Report

Title: Rapid development of cloud-native intelligent data pipelines for scientific data streams using the HASTE Toolkit

Version: Original Submission **Date:** 10/6/2020

Reviewer name: Brendan Lawlor

Reviewer Comments to Author:

The paper describes an approach to processing streams of scientific data in such a way as to maximise the use of limited available compute resources (cpu, storage and network bandwidth) by grading that data by "interestingness" and prioritizing data that is more interesting.
A number of git repositories is provided which together constitute a toolkit to facilitate this approach.
HASTE is the name of the toolkit, and will be used in this review also as the name for the overall approach.
The paper describes the essential characteristics of the approach, and presents two case studies to demonstrate how the toolkit might work in a scientific setting.
The two case studies outline the common overall flow of HASTE, but also its flexibility in catering to different needs of a pipeline (e.g. different resource bottlenecks, and different requirements with respect to latency).
The paper is well written and presents its case in a clear and readable fashion, although some sub-sections would benefit from additional attention in this regard.
The central concept of the HASTE approach, if correctly understood, is the separation of the Interestingness Function(IF) from the subsequent Policy that uses the IF to prioritize resources.
This strikes me as a sensible application of modularity to a scientific context, and would be likely to make the toolkit adaptable to different scenarios,
as indeed the case studies indicate.
Besides some minor proofreading corrections for grammar/readability which I would recommend, listed at the end, I would make two more significant observations and related recommendations, both related to reproducibility, and a final optional suggestion.
1. The body of the paper makes a good case for the IF/Policy split, but the Discussion and Conclusion sections seem undecided on what the reader should take away from the paper.
Should the reader attempt to reuse the HASTE toolkit, adapting it to their own needs? Or should they adopt the approach in the abstract, implementing it themselves?
Either (or indeed both) is acceptable, in my opinion, but the authors should be explicit in setting expectations for the reader about the viability of using the Toolkit.
In one paragraph, they describe HASTE as a "design pattern", which I think is a suitable term.
But in the following paragraph, they indicate their hope that the toolkit be "bolted on" to new and existing pipelines.
This would of course be valuable, but as a reader I would have appreciated some explicit direction on "next steps" if I found the pattern and toolkit potentially applicable to my work.

This would be a suitable outcome for a Technical Note.
The ability to use the toolkit hinges on various degrees of documentation and reproducibility.
While the code for the agent, report-generator, gateway etc are available on github, the README files appear aimed at the toolkit development team,
rather than potential users of the toolkit (or else too much is expected of those users).
If the authors intend to present the toolkit as a set of OSS resources for other researchers - which would be a worthwhile goal - it would be good to see a more
suitable documentation to guide, stepwise, those researchers. If this is the intent for the future, rather than as part of this publication, it would be helpful if the paper were to explicitly say so.
2. With regard to reproducibility of the experiments themselves, there are a few gaps that make it difficult for a reviewer,
even one who is familiar with AWS, k8s, docker and python, to reproduce and validate the results.
Kubernetes deployment descriptors are provided for the first case study, but the instructions are not clear
(for example actual resource instances are named, which will change with each deployment) and again seem for for internal use.
The instructions also presuppose the existence of a k8s cluster.
The data to be copied into the volumes is not provided.
The use of kubernetes is intelligent, as it is a de-facto standard in cloud computing, and there is an opportunity here for
researchers to make the experiments on such a cluster reproducible by providing details instructions for their assembly (using e.g. Terraform or AWS's CloudFormation),
or to provide an already-created cluster for that purpose,
and to provide the data on cloud storage that could be mapped to pods as part of the supplied kubernetes descriptors.
Note that the effort in implementing point 2 above would aid in realizing point 1.
For this reason, I would urge the authors to address this issue of experimental reproducibility. A reviewer should be able to:
a. Build all Docker images from source on the command line, pushing to an image registry
b. Follow clear stepwise instructions to deploy the images to a k8s cluster (either provided, or easily created using Terraform or Cloudformation)
c. Follow clear stepwise instructions to perform salient parts of the experiment from which the data was collected.
While this is not a trivial task, the result should lead to greater interest and uptake of the proposed model.
3. Finally, and entirely optionally, the authors may wish to give some consideration to the suitability of Functions as a Service (aka serverless, aka lambda functions) to implement the IF.
Minor corrections and suggestions follow:
1. Standardize the use of references: Where the reference is part of the sentence, use the authors name followed by the ref number. See first para on page 2, column 2 which first references Zhang et al, but then in the following sentence just uses [9] to reference Kelleher et al. There are a number of these inconsistencies in the paper.

2. Bottom of page 3. ", or send it" should probably be ",or if to send it" (or perhaps ", or whether to send it").
3. Page 4, column 1, para 3: "is that users' configure" - no need for apostrophe.
4. Page 6, column 1, para 2: Reword e.g. "image can have debris, can be out of focus..." etc.
5. Page 6, column 2, para 4: "which it to run" -> "which is to run".
6. Related to the above, the impression is given that k8s is a required component of the implementation. Apart from the issues of reproducibility of the *experimental data*, it would be good to be explicit with the reader on this matter (either here, or elsewhere in the paper).
7. Page 8, column 2, para 2: This section could do with a rewrite for clarity. I had difficulty understanding the meaning of "document index" in this context and this made interpretation of fig 5 problematic.
8. Page 8, column 2, final para: There appear to be two chained comparisons ("compared to", "when compared to") which renders the sentence confusing. In comparison to what is the 25% reduction in latency achieved?
9. Page 9, caption to figure 7: "is an artifact movement over the grid" -> "is an artifact of the movement over the grid"?
10. Page 9, Conclusion: There is an orphan closing parenthesis after the word "kinds".
11. Page 10, column 1, para 1: "creating an data hierarchy" -> "creating a data..."
12. Page 10, column 1 para 3: "self-explainable" -> "self-explanatory"?

Level of Interest

Please indicate how interesting you found the manuscript: Choose an item.

Quality of Written English

Please indicate the quality of language in the manuscript: Choose an item.

Declaration of Competing Interests

Please complete a declaration of competing interests, considering the following questions:

- Have you in the past five years received reimbursements, fees, funding, or salary from an organisation that may in any way gain or lose financially from the publication of this manuscript, either now or in the future?
- Do you hold any stocks or shares in an organisation that may in any way gain or lose financially from the publication of this manuscript, either now or in the future?
- Do you hold or are you currently applying for any patents relating to the content of the manuscript?
- Have you received reimbursements, fees, funding, or salary from an organization that holds or has applied for patents relating to the content of the manuscript?
- Do you have any other financial competing interests?

- Do you have any non-financial competing interests in relation to this paper?

If you can answer no to all of the above, write 'I declare that I have no competing interests' below. If your reply is yes to any, please give details below.

I declare that I have no competing interests

I agree to the open peer review policy of the journal. I understand that my name will be included on my report to the authors and, if the manuscript is accepted for publication, my named report including any attachments I upload will be posted on the website along with the authors' responses. I agree for my report to be made available under an Open Access Creative Commons CC-BY license (http://creativecommons.org/licenses/by/4.0/). I understand that any comments which I do not wish to be included in my named report can be included as confidential comments to the editors, which will not be published.

Choose an item.

To further support our reviewers, we have joined with Publons, where you can gain additional credit to further highlight your hard work (see: https://publons.com/journal/530/gigascience). On publication of this paper, your review will be automatically added to Publons, you can then choose whether or not to claim your Publons credit. I understand this statement.

Yes Choose an item.
