# Supplementary material for: Rapid development of cloud-native intelligent data pipelines for scientific data streams using the HASTE Toolkit
Source: Gigascience. 2021 Mar 19;10(3):giab018. doi: 10.1093/gigascience/giab018 (PMC7976223; doi:10.1093/gigascience/giab018)
Supplement: giab018_Reviewer_3_Report_Original_Submission — Beth Cimini -- 10/20/2020 Reviewed [file giab018_reviewer_3_report_original_submission.pdf]

Reviewer Report

Title: Rapid development of cloud-native intelligent data pipelines for scientific data streams using the HASTE Toolkit

Version: Original Submission **Date:** 10/20/2020

Reviewer name: Beth Cimini

Reviewer Comments to Author:

Overall, the work presented here represents an interesting approach to processing prioritization in biological imaging experiments, a generally neglected area. The work is well explained and is worthy of publication. I find particularly valuable the idea of separating out the interestingness function (IF) from the rest of the workflow, with the idea that the domain experts (biologists) can best assess what features make an image most interesting, and that the rest of the workflow can then be configured by a computational specialist able to prioritize and configure the (many!) separate moving parts of this workflow. Allowing the domain expert a particular place in the workflow to create systems that replicate their expertise can only lead to improved processing. Overall, it is a clever hybrid strategy and I can imagine many useful cases for it.

My major concern with the work presented (which is not a publication concern, but a longevity/usefulness concern) is that, while sufficiently documented for experts and "tinkerers", it is woefully under-documented for anyone who is not. The GitHub organization contains a variety of repositories, and it is not at all clear without reading this paper how they are meant to fit together with each other or with other packages; even after reading this paper, it is not clear how one would do any other activity besides those outlined in the authors case studies. The paper under consideration here is a good first step, but much more effort towards a consolidated package, or at least consolidated comprehensive documentation (ideally at or near where the code lives, IE on GitHub, or on the project website) will be very needed in order to gain any sort of adoption. This is particularly true for the kinds of models that can serve as the interestingness function- most domain experts would need significant guidance on how to turn their expertise into a function that fulfills the requirements of the HASTE system, and very little is provided. I do not think the work must be held for publication until this documentation appears, since it appears to be sufficiently documented for an expert to begin configuration, but it would be recommendation to the authors to prioritize such work.

Minor issue:

The authors state they use HasteStorageClient v0.13- their GH repo only reflects versions up to 0.10, indicating either commits are not being shared online or the authors need to be more careful with their versioning.

Level of Interest

Please indicate how interesting you found the manuscript: Choose an item.

Quality of Written English

Please indicate the quality of language in the manuscript: Choose an item.

Declaration of Competing Interests

Please complete a declaration of competing interests, considering the following questions:

- Have you in the past five years received reimbursements, fees, funding, or salary from an organisation that may in any way gain or lose financially from the publication of this manuscript, either now or in the future?
- Do you hold any stocks or shares in an organisation that may in any way gain or lose financially from the publication of this manuscript, either now or in the future?
- Do you hold or are you currently applying for any patents relating to the content of the manuscript?
- Have you received reimbursements, fees, funding, or salary from an organization that holds or has applied for patents relating to the content of the manuscript?
- Do you have any other financial competing interests?
- Do you have any non-financial competing interests in relation to this paper?

If you can answer no to all of the above, write 'I declare that I have no competing interests' below. If your reply is yes to any, please give details below.

I declare that I have no competing interests

I agree to the open peer review policy of the journal. I understand that my name will be included on my report to the authors and, if the manuscript is accepted for publication, my named report including any attachments I upload will be posted on the website along with the authors' responses. I agree for my report to be made available under an Open Access Creative Commons CC-BY license (http://creativecommons.org/licenses/by/4.0/). I understand that any comments which I do not wish to be included in my named report can be included as confidential comments to the editors, which will not be published.

Choose an item.

To further support our reviewers, we have joined with Publons, where you can gain additional credit to further highlight your hard work (see: https://publons.com/journal/530/gigascience). On publication of this paper, your review will be automatically added to Publons, you can then choose whether or not to claim your Publons credit. I understand this statement.

Yes Choose an item.
